# Low Complexity HEVC Encoder for Visual Sensor Networks

**DOI:** 10.3390/s151229788

**Published:** 2015-12-02

**Authors:** Zhaoqing Pan, Liming Chen, Xingming Sun

**Affiliations:** 1 School of Computer and Software, Jiangsu Engineering Center of Network Monitoring, Nanjing University of Information Science and Technology, Nanjing 210044, China; leegend_chen@outlook.com (L.C.); sunnudt@163.com (X.S.); 2 School of Computer Science and Engineering, Hebei University of Technology, Tianjin 300401, China

**Keywords:** visual sensor networks, video compression, HEVC, coding unit, low complexity

## Abstract

Visual sensor networks (VSNs) can be widely applied in security surveillance, environmental monitoring, smart rooms, *etc*. However, with the increased number of camera nodes in VSNs, the volume of the visual information data increases significantly, which becomes a challenge for storage, processing and transmitting the visual data. The state-of-the-art video compression standard, high efficiency video coding (HEVC), can effectively compress the raw visual data, while the higher compression rate comes at the cost of heavy computational complexity. Hence, reducing the encoding complexity becomes vital for the HEVC encoder to be used in VSNs. In this paper, we propose a fast coding unit (CU) depth decision method to reduce the encoding complexity of the HEVC encoder for VSNs. Firstly, the content property of the CU is analyzed. Then, an early CU depth decision method and a low complexity distortion calculation method are proposed for the CUs with homogenous content. Experimental results show that the proposed method achieves 71.91% on average encoding time savings for the HEVC encoder for VSNs.

## 1. Introduction

Visual sensor networks (VSNs) have emerged in response to the developments in image sensor technology, sensor networking and distributed computing, which have been widely used in security surveillance, environmental monitoring, smart rooms, and so on [1,2]. VSNs consist of a number of visual sensor nodes named camera nodes, which are used to collect visual information. However, the visual sensors generate a huge amount of visual data as compared to the traditional scalar sensors [3,4]. This becomes a challenge for storage, processing and transmitting the visual data due to the current storage, computing and transmission capability being still limited. Hence, the high compression rate and low complexity are the key requirements of VSNs [5,6]. High efficiency video coding (HEVC) [7,8] is the state-of-the-art video compression standard, which can efficiently compress the raw visual data. The compression rate of HEVC is twice as much as the former video compression standard, H.264 [9,10]. However, the achieved compression rate is at the cost of the heavy computational complexity of a series of advanced coding tools used in the HEVC, such as the quadtree-based coding unit (CU), the rate distortion optimization (RDO) technique, and so on. Hence, reducing the encoding complexity becomes vital for the HEVC encoder to be used in VSNs.

To reduce the encoding complexity of the HEVC encoder, many researchers have devoted their efforts to optimizing the HEVC encoding process [11,12]. In [11], Choi *et al.* proposed an early termination for the CU size decision process, in which the CU size decision process is terminated if the current CU selects the Merge/Skip mode as its best prediction unit mode. In [13], Pan *et al.* proposed a fast CU depth decision method by using the CU depth selection correlation between the current CU and its spatiotemporal neighboring CUs. In [14], Shi *et al.* proposed a fast CU size decision method based on adaptive CU depth selection, in which an adaptive CU depth set is derived by using the encoding information of the previously encoded frames. In [15], Zhang *et al.* proposed a machine learning-based coding unit depth decision for flexible complexity allocation in HEVC. Based on the spatiotemporal encoding parameters of the HEVC encoder, Ahn *et al.* proposed a fast CU encoding method, which consists of an early CU Skip mode decision method and a fast CU size decision method [16]. Based on the rate distortion differences between the root CU and children CUs, Goswami *et al.* proposed an early termination method for the CU depth decision process [17]. Based on the texture complexity of the video content, Tian *et al.* proposed an adaptive prediction unit mode decision for the HEVC intra-coding [18]. In [19], Kim *et al.* proposed a fast Skip mode decision method based on the rate distortion optimization for HEVC. According to the differential motion vector and coded block flag, an early determination of the prediction unit mode decision for HEVC was proposed in [20]. In [21], Lee *et al.* proposed an early Skip mode decision for the HEVC encoder by utilizing the rate distortion characteristics of the Merge mode. By considering the motion activity and hierarchical depth correlation, Pan *et al.* proposed an early Merge/Skip decision method for the low complexity HEVC encoder [12]. These methods can efficiently remove the encoding complexity of the HEVC encoder for universal video coding. However, the content characteristics of the videos that are generated by the visual sensor camera are not considered, and the encoding complexity can be further improved by considering the content property of the visual sensor videos.

In this paper, we propose a low complexity HEVC encoder for VSNs by optimizing the CU size decision process. The rest of this paper is organized as follows. Section 2 presents the motivations and statistical analyses. Section 3 introduces the details of the proposed fast CU size decision method. Experimental results are given in Section 4. At last, Section 5 concludes this paper.

## 2. Motivations and Statistical Analyses

**Figure 1:**
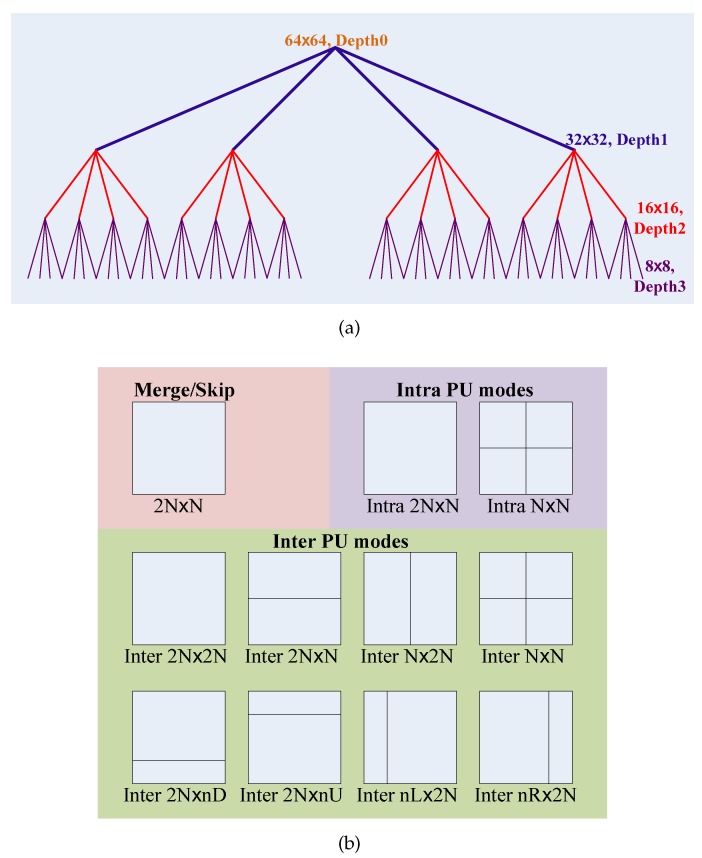
The quadtree-based coding units (CUs) and their prediction unit (PU) modes. (**a**) HEVC CU quadtree partition process; the maximum CU size = 64 × 64, the maximum quadtree depth = 4; (**b**) all prediction unit modes for a CU.

When compressing the raw videos that are generated by the visual sensor camera, the videos are separated into images/frames, then the images/frames are encoded one by one. Each frame is split into slices; the slices are further partitioned into a group of coding tree units (CTUs), which are the basic processing units of the HEVC encoder. Based on the quadtree, the CTUs are further split into CUs. In order to achieve the maximum compression rate, HEVC supports flexible CU sizes from 64 × 64 to 8 × 8, which corresponds to the CU quadtree Depth 0 to Depth 3. In the CU encoding process, the HEVC encoder checks $4^{n}$, *n*∈[0, 1, 2, 3], CU partitions for each quadtree depth level *n*, which totally equals $4^{0}+4^{1}+4^{2}+4^{3}=85$ CU partitions. For the CU intra-/inter-prediction, a CU is further partitioned into one, two or four prediction units (PUs) according to the prediction type, and the PU is the basic processing unit of the intra-/inter-prediction. To remove the spatial and temporal redundancy, the HEVC encoder supports 11 PU modes, including Merge/Skip mode, eight inter-PU modes (*i.e.*, inter-2N × 2N, inter-2N × N, inter-N × 2N, inter-N × N, inter-2N × nU, inter-2N × nD, inter-nL × 2N and inter-nR × 2N.) and two intra-PU modes (*i.e.*, intra-2N × 2N and intra-N × N.). For CU encoding, these 11 PU modes are checked sequentially. Figure 1 shows an example of the quadtree-based CUs and their PU modes. Ultimately, the best CU quadtree depth level, $d^{*}$, and the best PU mode, $p^{*}$, are determined according to the minimization of the Lagrangian cost function [22],

(1) $$\left\{\begin{array}{c} \left\{d^{*},p^{*}\right\}={arg min}_{d\in D}\left(\sum {arg min}_{p\in P}J\left(O,C(d,p)\right)\right)\\ J\left(O,C(d,p)\right)=SSD\left(O,C(d,p)\right)+\lambda \cdot R\left(d,p\right) \end{array}\right.$$

where **D** is the candidate set of the CU quadtree depth levels and D∈{0,1,2,3}; **P** indicates the candidate set of the 11 PU modes; *J* is the rate distortion cost function; ***O*** is the original CU; and ***C*** denotes the reconstructed CU, which is achieved by encoding the original CU ***O*** with the CU quadtree depth level *d* and PU mode *p*; the *SSD*(*d*,*p*) denotes the sum of squared differences (SSD) between the original CU ***O*** and its reconstructed CU ***C***; *λ* is the Lagrangian multiplier; *R*(*d,p*) represents the number of total bits for encoding this CU, which is obtained by a table lookup. This “try all and select the best” CU quadtree depth level decision and PU mode selection method efficiently improves the coding efficiency of the HEVC encoder; however, it also results in heavy computation load and limits the use of the HEVC encoder in VSNs.

In the CU encoding process, only parts of the CU partitions are finally chosen as the best CU partitions, which are from 1 to 64; “1” represents that the CU quadtree depth Level 0 is selected as the best depth level; “64” indicates the CU quadtree depth Level 3 is chosen as the optimal depth level. To analyze the best CU quadtree depth level distribution, three different HD visual sensor video sequences with a resolution of 1280 × 720, including “Fourpeople”, “Johnny” and “Vidyo1”, are tested. The HEVC reference software HM12.0 [23] is used as the software platform. The test conditions are listed as follows: the maximum CU size is 64 × 64, and the maximum CU quadtree depth is four; the fast motion estimation method is TZSearch, and the search range equals [−64, 64]; four quantization parameters (QPs) are adopted. The statistical results are tabulated in Table 1.

From Table 1, it can be observed that there are 68.26%, 16.07%, 14.33% and 1.34% CUs selecting the quadtree depth Levels 0, 1, 2 and 3 as their best CU quadtree depth level, respectively. In addition, the probability decreases as the depth level increases and as the QP decreases. This is because for the video that is generated by the visual sensor camera, it contains a huge number of regions with simple content, such as the background, and these regions are quite suitable for encoding in a large CU size. Moreover, more prediction residuals are transformed and quantized into zeros as QP increases, which results in the video content becoming simple, and more CUs select the large CU size as their best depth level. On the other hand, the CUs with depth Level 0 hold the largest proportion, and the number of CUs with depth Level 3 is quite small. Therefore, if the best quadtree depth level of a CU is determined early, significant encoding time could be saved.

**Table 1:** Statistical results of the best CU quadtree depth level distribution (%). QP, quantization parameter.

Sequence	QP	Level 0	Level 1	Level 2	Level 3
FourPeople	22	49.48	24.79	22.13	3.60
	27	66.59	16.50	15.19	1.71
	32	74.69	11.88	12.46	0.98
	37	79.57	8.78	11.15	0.50
Johnny	22	51.30	26.48	19.91	2.31
	27	68.30	17.63	13.26	0.81
	32	76.74	11.87	10.95	0.44
	37	82.57	7.58	9.59	0.25
Vidyo1	22	48.94	26.55	21.07	3.44
	27	65.94	18.27	14.51	1.27
	32	74.66	13.12	11.66	0.55
	37	80.34	9.35	10.07	0.24
Average		68.26	16.07	14.33	1.34

## 3. The Proposed Low Complexity HEVC Encoder for VSNs

### 3.1. The Proposed All-Zero Block-Based Fast CU Depth Decision Method

**Figure 2:**
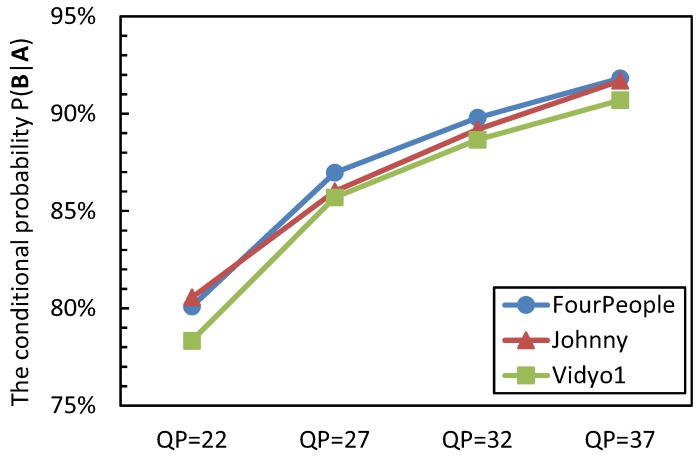
The conditional probability P(**B**|**A**).

The videos that are captured by the visual sensor camera contain a huge number of homogenous regions, such as the background; for these regions, they are quite suitable for encoding in a large CU partition size due to a large-sized CU being able to represent the prediction residual in a small number of symbols than is possible in the case of several small-sized CUs [24]. In addition, the inter-prediction residuals of these regions have a large probability to be transformed and quantized to zeros [25,26,27]. Therefore, it is reasonable to design an early termination for the CU size decision process based on the quantized coefficients. In video coding, the CU for which its inter-prediction/intra-prediction residual is transformed and quantized to zeros is called the all-zero block (AZB). To exploit the relationship between the AZB and the best CU depth selection, the event **A** indicates after encoding the CUs of depth level *i* with the Megre/Skip mode and inter-2N × 2N PU mode; they are AZBs. The event **B** represents that the CU quadtree depth *i* is chosen as the best CU depth level; the conditional probability P(**B**|**A**) is analyzed. The statistical results are shown in Figure 2.

From Figure 2, it can be seen that when the CUs with quadtree depth *i* are AZBs, the CU has a rather large probability to select the quadtree depth level *i* as its optimal depth level. The conditional probability of *P*(**B**|**A**) is from 78.34% to 91.82%, 86.63% on average. It also can be observed that the probability of *P*(**B**|**A**) increases as the QP value becomes large; this is because the large QP makes the encoding content become simple and homogenous, which results in more prediction redials being quantized to zeros. Hence, based on the above analyses, the optimal CU quadtree depth level decision process is terminated early if:

(2) $$\sum_{i}{Q_{Merge}}_{i}+\sum_{i}{Q_{Inter2N\times 2N}}_{i}=0$$

where the *i* represents the number of total CUs, *i* = $4^{n}$, n∈[0,1,2,3], *n* is the quadtree depth level; ${Q_{Merge}}_{i}$ is equal to zero, if the prediction residuals of the Merge/Skip mode are quantized to zeros, otherwise, ${Q_{Merge}}_{i}$ is larger than zero; ${Q_{Inter2N\times 2N}}_{i}$ is equal to zero, if the prediction residuals of the inter-2N × 2N mode are quantized to zeros, otherwise, ${Q_{Inter2N\times 2N}}_{i}$ is larger than zero.

### 3.2. Efficient Distortion Estimation Based on Spatial Correlation

In the inter-prediction process, the distortion value is used to find the best matching block. The sum of absolute distortion (SAD) is a normal distortion calculation method in video coding; however, the distortion calculation process consumes the majority of the total encoding time [22]. If this process can be simplified, much more of the encoding time could be reduced. In the visual sensor videos, there exist a huge number of homogenous regions, such as the background. In this paper, the content property of one CU is determined according to the quantized coefficients; thus, the current encoding CU belongs to a homogenous region if:

(3) $$Q_{Merge}+Q_{Inter2N\times 2N}=\text{0}$$

where $Q_{Merge}$ denotes the quantized coefficients of the CU, which is encoded with the Merge/Skip mode, if after encoding the CU with the Merge/Skip mode, the prediction residuals are transformed and quantized to zeros, then the value of $Q_{Merge}$ is equal to zero; $Q_{Inter2N\times 2N}$ indicates the quantized coefficients of the CU, which is encoded with the inter-2N × 2N mode; the value of $Q_{Inter2N\times 2N}$ equals zero, if the prediction residuals are transformed and quantized to zeros after encoding the CU with the inter-2N × 2N mode.

**Figure 3:**
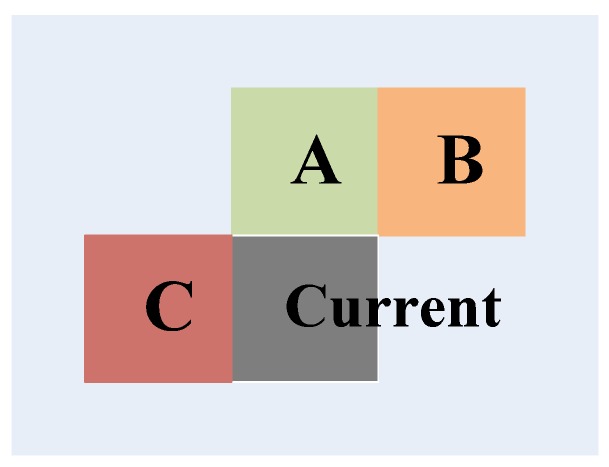
The spatial neighboring blocks of the current encoding block.

Additionally, there is rather high distortion correlation in the spatial domain for these homogenous regions. Figure 3 shows an example of the spatial neighboring blocks of the current encoding block, where A, B and C represent the spatial neighboring blocks on the up, up-right and left of the current encoding block, respectively. Based on the spatial correlation [28,29], the motion vector of the current block, $M{\vec{V}}_{Pred}$, is predicted by the spatial neighboring blocks and is given in Equation (4),

(4) $$M{\vec{V}}_{Pred}=\text{Median}\left\{M{\vec{V}}_{up},MV_{u\vec{p}-right},M{\vec{V}}_{left}\right\}$$

where $M{\vec{V}}_{up}$, $MV_{u\vec{p}-right}$ and $M{\vec{V}}_{left}$ indicate the motion vector of the neighboring blocks of up, up-right and left of the current block. Then, the predictive distortion for the CU with homogenous content, $SAD_{Pred}$, is obtained by:

(5) $$SAD\left(o,c\left(M{\vec{V}}_{Pred}\right)\right)=\sum_{x=1}^{P_{x}}\sum_{y=1}^{P_{y}}\left|o\left(x,y\right)-c\left(x-{MV_{Pred}}_{x},y-{MV_{Pred}}_{y}\right)\right|$$

where $P_{x}$ and $P_{y}$ mean the prediction unit size; *o* is the current value, and *c* indicates the reconstructed block value; $M{\vec{V}}_{pred}$=$\left({Pred}_{x},{Pred}_{y}\right)$ is the predictive motion vector.

**Figure 4:**
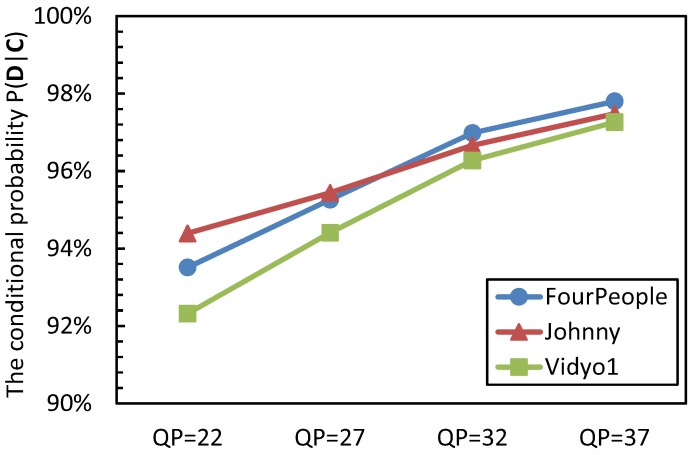
The conditional probability P(**D**|**C**).

To evaluate the distortion estimation accuracy, the event **C** represents that the current PU belongs to a homogenous region, and the event **D** denotes that the distortion that is obtained by Equation (4) and Equation (5) is equal to the value that is gained by the original motion estimation. The conditional probability of *P*(**D**|**C**) is analyzed, and the results are shown in Figure 4.

From Figure 4, it can be seen that the CU belongs to the homogenous region, and there is a large probability that the predictive distortion is the same as the distortion obtained by the original motion estimation. The probability of *P*(**D**|**C**) is from 92.32% to 97.80%, 95.65% on average. We can also see that with the increase of QP values, the value of *P*(**D**|**C**) becomes larger; this is because the large QP makes the coding content become homogenous. From these values, we can draw the conclusion that the proposed distortion estimation method can efficiently predict the distortion for a CU with homogenous content.

### 3.3. The Overall Algorithm

Based on the above analyses, the proposed fast CU size decision method for the low complexity HEVC encoder is summarized in Algorithm 1.
 **Algorithm 1** Proposed fast CU size decision method for the low complexity H.265/HEVC encoder.   **Input**: CTU size = 64 × 64, the maximum quadtree depth level = 4   **for**
*Depth level*=0 to 3 **do**    Encode the current CU with the Merge/Skip mode    Encode the current CU with the inter-2N×2N mode    **if**
$Q_{Merge}+Q_{Inter2N\times 2N}=\text{0}$
**then**     The predictive distortion of the remaining inter-prediction modes is obtained by Equation (4) and Equation (5)    **else**     The predictive distortion of the remaining inter-prediction modes is achieved by the original motion estimation    **end**
**if**    **if**
$\sum_{i}{Q_{Merge}}_{i}+\sum_{i}{Q_{Inter2N\times 2N}}_{i}=0$
**then**     The CU size decision process is terminated    **else**
     Encode the current CTU with the next quadtree depth level    **end**
**if**    **Output**: The best CU quadtree depth level   **end**
**for**   Process the next CTU

## 4. Experimental Results

**Figure 5:**
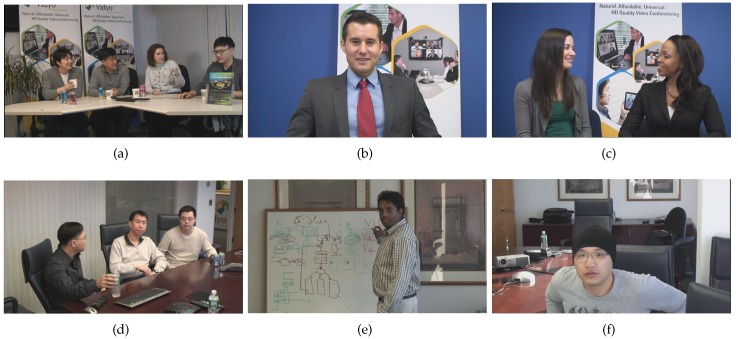
The test sequences. (**a**) FourPeople; (**b**) Johnny; (**c**) KristenAndSara; (**d**) Vidyo1; (**e**) Vidyo3; (**f**) Vidyo4.

To evaluate the efficiency of the proposed fast CU size decision method, the HEVC reference software HM12.0 is used as the software platform. The test conditions are listed as follows: the maximum CU size is 64 × 64; the maximum CU quadtree depth level is four; the motion estimation method is TZSearch, and the search range equals [−64, 64]; four QPs, 22, 27, 32 and 37, are used in our experiments. Six visual sensor video sequences, including “FourPeople”, “Johnny”, “KristenAndSara”, “Vidyo1”, “Vidyo3” and “Vidyo4”, are adopted. These six video sequences are shown in Figure 5. The detailed information of these six sequences is that the resolution is 1280 × 720; the frame rate equals 60 fps; the number of encoded frames is 193. The hardware platform is Intel Xeon CPU E3-1241 v3 with 3.50 GHz and 3.50 GHz, 4.00 GB RAM with the Microsoft Windows 7 64-bit operating system.

We compared the encoding performance of the proposed method with Choi [11], Kim [20] and Pan [12], in terms of peak signal-to-noise ratio (PSNR), bit rate (BR) and total encoding time savings. The experimental results are summarized in Table 2. In the table, Δ*T* means the total encoding CPU time savings, and this is computed as:

(6) $$\left\{\begin{array}{c} \Delta PSNR=PSNR_{\Omega }-PSNR_{o},\\ \Delta BR=\frac{BR_{\Omega }-BR_{o}}{BR_{o}}\times 100\%,\\ \Delta T=\frac{T_{\Omega }-T_{o}}{T_{o}}\times 100\% \end{array}\right.$$

where $PSNR_{\Omega }$, $BR_{\Omega }$ and $T_{\Omega }$ representing the PSNR, BR and total encoding time of method Ω; Ω∈{Choi, Kim, Pan}, $PSNR_{o}$, $BR_{o}$ and $T_{o}$ indicate the PSNR, BR and total encoding time of the original HM12.0. In addition, the average Δ*PSNR* and Δ*BR* are computed by the Bjontegaard delta PSNR (BDPSNR) and Bjontegaard delta BR (BDBR) [30,31], respectively.

From Table 2, it can be seen that Choi’s method reduces the total encoding time from 32.54% to 70.17%, 55.47% on average. Meanwhile, the PSNR degrades from 0.022 dB to 0.071 dB, and the BR increases from −0.38% to −1.99%. The average PSNR variation is from −0.003 dB to 0.033 dB, 0.002 dB on average, and the average BR changes from −0.98% to 0.70%, 0.07% on average. Kim’s method saves the total encoding time from 29.06% to 56.22%, 45.71% on average. At the same time, the average PSNR change is from −0.025 dB to 0.030 dB, −0.005 dB on average, and the average BR variation is from −0.90% to 0.68%, 0.18% on average. Pan’s method improves the encoding complexity from 33.82% to 69.12%, 54.28% on average. Meanwhile, the average PSNR degrades from 0.009 dB to 0.031 dB, 0.018 dB on average, and the BR increases from 0.34% to 1.15%, 0.67% on average. The proposed method reduces the total encoding time from 51.27% to 85.26%, 71.91% on average. Moreover, the PSNR degrades from 0.030 dB to 0.114 dB, and the BR variation is from −1.43% to 0.70%. The average PSNR change is from −0.064 dB to 0.033 dB, −0.021 dB on average, and the average BR variation is from −1.02% to 1.98%, 0.73% on average. From these values, we can observe that the proposed method achieves a similar rate distortion performance as these compared methods, while the encoding complexity saving performance is the best among these compared methods.

**Figure 6:**
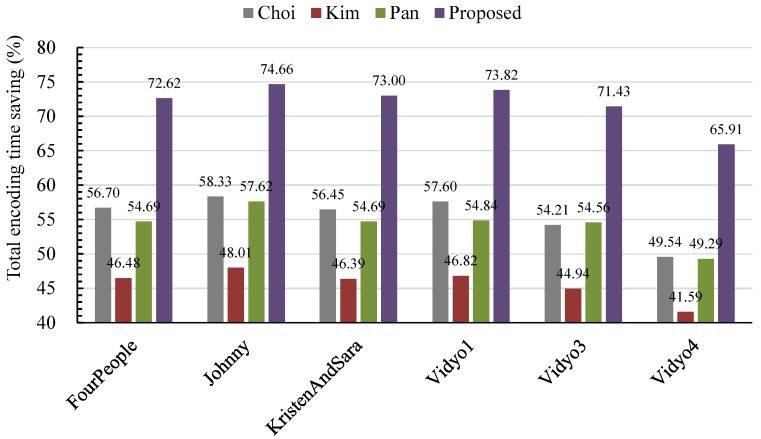
Comparison of the total encoding time savings.

To intuitively show the encoding time saving performance of the proposed method, the encoding time saving comparison among the Choi, Kim, Pan and proposed method is given in Figure 6. It can be observed that the proposed method obtains the best complexity saving performance. Compared to Chio’s, Kim’s and Pan’s methods, the proposed method reduces 36.92%, 48.26% and 38.56% of the total encoding time, respectively. These values demonstrate that the proposed method works efficiently for reducing the encoding complexity of the HEVC encoder for VSNs.

**Table 2:** Summary of the encoding results. BR, bit rate.

Sequence	QP	Choi [11] *vs.* HM			Kim [20] *vs.* HM			Pan [12] *vs.* HM			Proposed *vs.* HM		
		ΔPSNR	ΔBR	ΔT	ΔPSNR	ΔBR	ΔT	ΔPSNR	ΔBR	ΔT	ΔPSNR	ΔBR	ΔT
		(dB)	(%)	(%)	(dB)	(%)	(%)	(dB)	(%)	(%)	(dB)	(%)	(%)
FourPeople	22	−0.030	−1.32	−41.84	−0.008	0.07	−35.70	−0.014	−0.08	−39.27	−0.034	−1.13	−58.04
	27	−0.039	−1.28	−55.15	−0.013	−0.41	−45.36	−0.027	0.15	−52.35	−0.041	−0.99	−71.59
	32	−0.025	−0.65	−62.45	0.001	−0.12	−50.67	−0.029	0.81	−60.81	−0.047	−0.57	−78.38
	37	−0.029	−0.62	−67.37	−0.012	0.04	−54.20	−0.019	−0.38	−66.34	−0.063	0.06	−82.46
	Average	−0.004	0.09	−56.70	−0.002	0.06	−46.48	−0.011	0.34	−54.69	−0.021	0.61	−72.62
Johnny	22	−0.024	−1.22	−41.67	−0.010	−0.52	−36.45	−0.019	−0.32	−39.61	−0.032	−1.04	−58.17
	27	−0.030	−1.16	−56.50	−0.014	−0.21	−46.92	−0.016	−0.57	−56.67	−0.047	−1.43	−74.11
	32	−0.046	−1.21	−64.98	−0.029	−0.35	−52.46	−0.042	−0.53	−65.08	−0.064	−1.62	−81.09
	37	−0.039	−1.30	−70.17	−0.026	−0.43	−56.22	−0.021	−0.38	−69.12	−0.075	−1.26	−85.26
	Average	−0.003	0.22	−58.33	−0.009	0.44	−48.01	−0.008	0.57	−57.62	−0.017	0.69	−74.66
KristenAndSara	22	−0.035	−1.12	−41.23	−0.010	−0.28	−35.41	−0.014	−0.08	−39.27	−0.047	−1.08	−57.91
	27	−0.047	−1.44	−54.02	−0.014	−0.09	−44.77	−0.027	0.15	−52.35	−0.072	−2.35	−71.39
	32	−0.050	−1.99	−62.69	−0.013	0.00	−50.86	−0.029	0.81	−60.81	−0.079	−1.59	−79.14
	37	−0.053	−1.27	−67.84	−0.012	0.19	−54.51	−0.019	−0.38	−66.34	−0.089	−0.84	−83.58
	Average	−0.004	0.01	−56.45	−0.012	0.39	−46.39	−0.031	1.15	−54.69	−0.019	0.68	−73.00
Vidyo1	22	−0.034	−1.40	−43.63	−0.003	0.21	−36.67	−0.014	−0.36	−40.42	−0.042	−1.51	−59.66
	27	−0.042	−1.28	−55.67	−0.013	−0.22	−45.59	−0.026	−0.82	−52.61	−0.056	−0.78	−72.37
	32	−0.043	−1.06	−62.99	−0.024	−0.46	−50.79	−0.027	−0.40	−61.17	−0.063	−0.57	−79.38
	37	−0.036	−1.40	−68.12	−0.022	−0.76	−54.25	−0.023	0.12	−65.18	−0.070	−0.64	−83.88
	Average	0.033	−0.98	−57.60	0.030	−0.90	−46.82	−0.009	0.29	−54.84	0.033	−1.02	−73.82
Vidyo3	22	−0.038	−0.80	−40.47	−0.010	−0.16	−34.68	−0.017	−0.29	−39.41	−0.053	−1.11	−57.07
	27	−0.045	−0.69	−51.98	−0.020	0.06	−43.51	−0.025	−0.05	−51.86	−0.061	−0.40	−69.79
	32	−0.059	−0.84	−59.36	−0.041	−0.30	−49.01	−0.036	0.52	−60.94	−0.114	−0.39	−76.91
	37	−0.071	−0.75	−65.03	−0.017	0.69	−52.55	−0.034	0.39	−66.03	−0.112	0.16	−81.96
	Average	−0.024	0.70	−54.21	−0.025	0.68	−44.94	−0.032	1.04	−54.56	−0.064	1.98	−71.43
Vidyo4	22	−0.029	−0.69	−32.54	−0.008	−0.09	−29.06	−0.015	−0.26	−33.82	−0.030	−0.72	−51.27
	27	−0.022	−0.72	−46.57	−0.009	−0.20	−39.92	−0.024	−0.14	−47.37	−0.037	−0.58	−57.46
	32	−0.026	−0.38	−56.17	−0.011	0.29	−46.56	−0.016	−0.03	−55.37	−0.041	0.70	−74.86
	37	−0.028	−0.40	−62.89	−0.009	0.03	−50.81	−0.017	0.03	−60.62	−0.049	0.46	−80.07
	Average	−0.009	0.39	−49.54	−0.008	0.38	−41.59	−0.018	0.60	−49.29	−0.036	1.46	−65.91
Average		−0.002	0.07	−55.47	−0.005	0.18	−45.71	−0.018	0.67	−54.28	−0.021	0.73	−71.91

## 5. Conclusions

High computational complexity becomes a bottleneck for the HEVC encoder to be used in VSNs. In order to reduce the encoding complexity of the HEVC encoder, in this paper, we proposed a low complexity HEVC CU size decision method based on the quantized coefficients, which consists of a CU depth early termination method and a low complexity distortion calculation method. Experimental results show that the proposed method can efficiently reduce the encoding complexity of the HEVC encoder of VSNs.
